# Aggressive behaviour during a standardized play observation in 3 to 6-year-old children with and without refugee experience: an observational study

**DOI:** 10.1186/s13034-026-01071-y

**Published:** 2026-03-27

**Authors:** Ana Mihaljevic, Jan Justus Kuther, Monika Fink, Maria Licata-Dandel, Volker Mall, Andrea Hahnefeld

**Affiliations:** 1 TUM School of Medicine and Health, Chair of Social Pediatrics, Technical University of Munich (TUM), Munich, Germany; 2 Kbo Kinderzentrum, Heiglhofstraße 69, 81377 Munich, Germany

**Keywords:** Refugee, Children, Aggressive behaviour, Play observation, Trauma, Screen time

## Abstract

**Background:**

Exposure to refugee and traumatic experiences in early childhood can dysregulate the stress response system and lower children’s threshold for aggression. This study examines differences in amount and patterns of aggressive play in children with and without refugee or traumatic experiences, while also assessing how other environmental factors, such as flight duration as a marker of cumulated adverse experiences, time spent in the host country, parental distress, and screen exposure, are associated with such behaviour.

**Methods:**

In standardized 10 min free individual play sessions, episodes of aggressive behaviours were observed in 62 children with refugee experience and 64 children from a clinically referred comparison group without refugee experience (aged 3–6 years) and coded into four categories: physical, verbal, instrumental, and symbolic. Emotional states were documented to clarify the background mood and context of the aggressive episodes. Parents reported sociodemographic data, screen time, and filled out the Child and Adolescent Trauma Screening for children aged 3–6 (CATS 3–6), assessing potentially traumatic experiences of their children with corresponding symptoms, and the Refugee Health Screener (RHS) for information on their own mental health and distress levels. We correlated these factors with our structured observations of aggressive episodes during children’s play in a standardized situation.

**Results:**

No significant difference in the distribution of aggressive behaviour episodes between the refugee and comparison groups was observed, even after accounting for gender. Symbolic aggression was more frequent among boys from the comparison group (p = 0.014), while no group or gender effects emerged for other aggression categories. Amount of aggressive behaviour was not related to parent-rated traumatic experiences or symptoms, but a significant correlation appeared, with the reported amount of screen time within the male sub-group (ρ = 0.209, p =0 .027).

**Conclusion:**

Refugee and traumatic experiences showed no association with increased aggressive behaviour, whereas higher screen time was observed alongside elevated amounts of aggressive episodes among boys in our study. Children from both, refugee and clinical comparison group predominantly engaged in non-violent play in calm, low demand setting, highlighting potential protective role of safe, stimulating environments.

*Trial registration* German clinical trials register, registration number: DRKS00025734, date: 07–23-2021.

**Supplementary Information:**

The online version contains supplementary material available at 10.1186/s13034-026-01071-y.

## Background

The number of forcibly displaced children has increased markedly over the past decade, rising from approximately 18.8 million in 2010 to an estimated 47.2 million worldwide by the end of 2023 [1]. Germany, as one of the largest host countries [2], accommodates around 1.4 million children and adolescents under the age of 18 with refugee experiences [3]. They often face violence, abuse, life-threatening situations, loss of family members, and prolonged uncertainty during a sensitive developmental period [4]. Being exposed to such adversity can dysregulate the stress response system in children, leading to heightened irritability, anger, and patterns of defensive or aggressive behaviours, known as "survival states." [5, 6]. In line with this, in several studies, children with refugee experience displayed more problematic behaviours, including various forms of aggression, particularly physical aggression, compared to children without refugee experience [7–10]. However, other recent studies report no association between refugee status and excess of externalizing or overall behaviour problems and, in some cases, even similar or better mental health outcomes, including lower internalizing and externalizing symptoms, among children with refugee experience compared to ones without [11–13]. These findings suggest mixed evidence in this area and highlight importance of understanding potential mechanisms and environmental factors that may account for the presence or absence of an association between refugee status and behavioural escalations.

In early childhood, as children are still learning to regulate their emotions, express needs, and interact socially, aggression is often considered a normal and functional feature of development, in the context of exploring new behaviours [14–16]. Challenging and potentially traumatic experiences in early childhood can be associated with developmental problems and difficulties in emotional regulation. Children who struggle with emotional control are more likely to act physically aggressive, particularly in stressful situations [17, 18]. As a potential result, maladaptive behaviours, social problems, and higher levels of aggression might develop in children affected by early adversity [19–21]. In Pfeiffer et al. (2022), the trauma load during flight remained consistently high, indicating that flight is an important marker of adversity, associated with cumulative traumatic experiences [22]. For children growing up in such unstable environments, aggression can also serve as a coping mechanism for self-defence, as well as for fear and helplessness, which tends to escalate in the absence of co-regulation and proper support [23, 24]. From an attachment theory perspective, early adversity and prolonged instability may disrupt caregiver–child co-regulation processes, increasing the likelihood that children express distress through dysregulated or aggressive behaviours in challenging situations [25]. Therefore, extended flight duration and high trauma load can represent risk factors for children with refugee experience, potentially leading to more aggressive behaviour sequences in various situations.

At the same time, current living conditions, including relationships to caregivers and everyday activities, appear to be just as important. To learn how to handle strong emotions like frustration, anger, or fear, young children depend on experiences in their environment and sensitive reactions from their primary caregivers to learn to co-regulate [16]. Previous research indicates that parental mental health issues can indirectly influence the occurrence of aggressive or other externalizing behaviours in children [26]. As an example, the combination of stressful psychosocial factors, such as lower levels of parental education and higher stress, contributes to greater screen exposure in children, which in turn negatively impacts their cognitive development and stress regulation [27]. This excessive screen time, including both smartphones and television, has been associated with increased aggressive behaviour in young children [28–31]. Conversely, integration into supportive environments like school programs, peer groups, and/or community initiatives can often reduce aggressive behaviour and support better emotional adjustment [11, 32–34]. Together, these findings highlight the interplay of past experiences, family stress, and current environment in shaping the behaviour of children with refugee experience.

In addition to direct experiences, children often learn by watching others, a process known as observational learning. According to Bandura’s social learning theory, children acquire behaviours by watching their role models, storing observed behaviours in memory, and reproducing them when appropriate, especially if they perceive the behaviour as successful or rewarding [35, 36]. Observational learning mechanisms like imitation of violent models, desensitization to aggressive content, and priming of aggressive scripts explain how exposure to violence, whether real or in media, influences aggressive behaviour [37, 38]. Accordingly, previous literature suggests that children who witness violence are likely to develop patterns of hostile attributions and a tendency to respond aggressively, even to neutral cues [39]. These insights are important to understand how repeated exposure to violence during flight might be reinforcing aggressive behaviour patterns in refugee children.

While a child who feels safe may express conflict playfully through symbolic aggression [16], a child who feels threatened might react with physical violence [40, 41], showing that patterns (categories) of aggression may vary depending on a child's background. Symbolic aggression, characterized by pretend play where children simulate conflict scenarios without genuine hostility, has been described in previous studies as a part of sociodramatic play, essential for social and cognitive development, helping children to understand complex ideas about social roles, conflict, and cooperation [16, 42–44]. While often misunderstood by caregivers as hostile, this type of play is not typically associated with actual violence [3] and has even been positively linked to prosocial behaviour rather than physical aggression [45, 46]. It is important to differentiate between types of aggression and to understand that not all aggressive behaviour in children indicates real violence [16].

Aggression in children is commonly assessed through parent and teacher reports, such as the Strengths and Difficulties Questionnaire (SDQ), Child Behaviour Checklist (CBCL), and Buss-Perry Aggression Questionnaire (BPAQ) [47–50]. Although self-reports and parent ratings are widely used [7, 8], they are susceptible to biases, including social desirability [5]. Alternative approaches include observational methods, such as naturalistic or structured free-play observations in classrooms, playgrounds, and laboratory settings [51, 52]. Other studies employ the Play Observation Scale (POS) or adapted versions to track behavioural patterns over time, including aggression [53]. However, these tools often code aggression merely as present or absent, lacking subcategorizations or assessments of associated emotional states [54, 55].

Although observational methods are established in aggression research, no standardized culturally sensitive protocols specifically target children with refugee experiences [51]. Prior work highlights the utility of structured play observations in identifying behavioural patterns in this population, particularly in assessing play complexity, social interaction, and emotional detachment [19, 56, 57]. For instance, the INCLUDE group developed and validated an observation sheet to evaluate play behaviour during structured sessions, yet it still does not thoroughly assess aggression categories or intensity [19].

Overall, previous studies have highlighted the need for systematic, culturally sensitive observation of aggression in refugee children, focusing on both quantitative levels and qualitative forms. Our study addresses this need by observing aggressive behaviour in 3- to 6-year-old children with and without refugee experience during standardized play sessions. We distinguish between various categories of aggression and examine the influence of environmental and contextual factors, such as trauma exposure, flight duration, time since arrival in Germany, parental distress and education, and screen time, on levels of aggression. The predictors were selected to capture complementary domains relevant to early childhood development, including indicators of past adversity, current psychosocial context, and everyday environmental exposure. These factors have been linked to children’s emotional regulation and behavioural expression in previous research, and their combination reflects the multifactorial nature of aggressive expressions in early childhood rather than a single causal pathway. Accordingly, the present study adopts an exploratory approach to examine how these interrelated factors co-occur with different forms of aggressive expressions during the play. The following hypotheses were tested:

### H1a

Children with refugee experience display more episodes of aggressive behaviour in spontaneous play than children without refugee experience.

### H1b

The distribution of children in aggressive categories differs between children with and without refugee experience, with more physical aggression in the refugee group.

### H2

Children whose parents reported potentially traumatic experiences exhibit more episodes of aggressive behaviour during spontaneous play compared to children without reported potentially traumatic experiences.

### H3

 Greater exposure to environmental stressors (i.e., adverse experiences, longer flight duration, higher screen time, and parental distress) is associated with a higher prevalence of aggressive behaviour in play, whereas longer time since arrival in Germany and more years of parental education is associated with lower levels of such behaviour

## Methods

### Study design, procedure, and population

This cross-sectional study is part of the InterCuLtUral Child Development Project (INCLUDE) and presents a retrospective analysis of existing data and video material collected between June 2021 and August 2022.

Children with refugee experience were recruited and assessed in two refugee camps in Munich, Germany, as part of standardized developmental assessments. The families were informed about the study and participated voluntarily, with interpreters provided when needed. Inclusion criteria were age between 3 and 6 years and a documented refugee or flight background. Children with expert-diagnosed autism spectrum disorder, severe intellectual impairment, specific genetic syndromes, or serious neurological conditions were excluded in both groups.

The age-matched clinical comparison group comprised children born in Germany who were referred to the Social Pediatric Center (SPZ Schwabing, Munich) between January and April 2022 for assessment due to developmental, language, and/or socio-emotional difficulties. Within this group, 17 children (27%) were diagnosed with combined developmental disorders (F83), 20 children (31%) with speech or motor developmental disorders (F80, F82), and 30 children (47%) received psychiatric diagnoses (F43, F90–F98). This group represents a clinical comparison sample rather than a community-based population sample and has been used and described in our previous studies. For more detailed information on study participants and design, see Bernhardt et al. (2023) [19] and Hahnefeld/Fink et al. (2024) [27].

### Assessment

#### Context factors

Parents were asked to supply the socio-demographic data of the child, including flight duration and months spent in Germany. Additionally, they reported the child's total screen time per day, within one of these three categories:

$$\begin{aligned} 1 &= {\text{ }} < 1{\text{ }}hour/day; \\ 2 & = {\text{ }}1 - 2{\text{ }}hours/day; \\ 3 & = {\text{ }} > 2{\text{ }}hours{\text{ }}/day \\ \end{aligned} $$

Formal parental education (mean years of schooling of both parents) was examined as a proxy for socioeconomic status and correlated with aggressive behaviour. Most environmental predictors were analysed across the pooled sample to examine cross-group associations, given limited within-group variability and shared contextual characteristics between refugee and clinically referred children; parental education was analysed also separately due to expected group differences.

### Adverse experiences and posttraumatic stress

Parents were asked to complete the CATS 3–6 Questionnaire (Child and Adolescent Trauma Screening for children aged 3–6) [57], an instrument to evaluate exposure to potentially traumatic experiences and PTSD symptoms based on DSM-5 [58] criteria. In the first section, caregivers indicate categories of such experiences the child has faced. The second section focuses on the child’s symptoms, rating behaviours or emotional responses observed in the child, such as nightmares, being overly fearful, aggressive behaviour, avoidance of certain places or people, etc. The recommended cut-off score of ≥ 16 were considered indicative of clinically relevant level of stress symptoms. The CATS 3–6 has demonstrated good internal consistency and construct validity in trauma-exposed and clinically referred samples [57].

### Caregiver mental health

Parents of both groups filled out the RHS (Refugee Health Screener). These approved, cross-cultural screening tool captures symptoms accompanying psychiatric disorders, primarily designed with the help of persons with refugee experiences. The first 14 items assess trauma sequelae symptoms, with a cut-off score of ≥ 12 indicating elevated symptom load. The 15th item, a visual analog ‘‘distress thermometer’’, captures current perceived distress on a scale from 0 to 10, with a cut-off score of ≥ 5 defined as clinically relevant [59]. The RHS has shown excellent reliability and validity in refugee populations, while evidence in non-refugee samples remains limited [60]. In the present sample, the RHS-15 showed excellent internal consistency (Cronbach’s α = 0.95), indicating good reliability of the measure in this context.

### Play observation

Every child was offered the same pre-structured standardized play setting, including an animal farm, two dolls, cooking equipment, and a construction kit with multi-coloured wooden bricks. Children played alone and were allowed to start playing spontaneously without any prompts from examiners. Ten-minute-long play episodes were recorded (for further information, see Bernhardt et al. (2023) [19].

### Analysing and counting aggressive behaviours

Based on Buss et al., we developed a structured approach incorporating classificational and observational methods to analyse and count aggressive behaviours in video recordings [61]. The literature lacks a consistent and standardized differentiation of aggression subtypes, with fluid transitions and overlapping definitions across studies. [62]. Suitable for our single-child play setting [63], we differentiated between 4 categories: [64]

(A) Physical Aggression: Includes actions like throwing, hitting, or pushing objects [64, 65].
(B) Verbal Aggression: Rare in solitary play but often includes hostile remarks [61, 62].
(C) Instrumental Aggression: Goal-driven actions, like forceful manipulation of toys [66].
(D) Symbolic Aggression: Imaginative, playful enactments, such as toy battles [16].

For better illustration, symbolic aggression was coded when aggressive themes were enacted through pretend play without direct physical force, such as aiming a toy weapon at an animal figure or simulating fighting between toy characters. Physical aggression included behaviours such as forceful hitting or throwing of toys representing direct physical impact. Instrumental aggression referred to goal-directed actions aimed at obtaining objects, while verbal aggression captured hostile verbal expressions during the play.

Inspired by the time-sampling method of the Play Observation Scale (POS) [53], video recordings were segmented into 30 s intervals, with each category of aggression coded as present or absent per interval [67]. To contextualize the behaviour, children’s emotional state during aggressive episode was noted descriptively by the observer (e.g., frustrated, playful, etc.) as well as a short comment describing observed behaviour. This measure was not based on a validated coding system and was intended solely for exploratory, descriptive purposes. The intended duration of the video recording of the children's play was 10 min. However, as many of the videos were either shorter or longer than this, the absolute number of aggressive episodes observed was corrected to account for the differences in video length. The ratings of the videos were conducted blindly concerning the backgrounds of the children. All video material was coded by a single researcher using a theory-driven coding scheme developed for the present study. In cases of ambiguity, coding decisions were discussed with a senior researcher to ensure consistency with the conceptual framework. No formal observer training sessions or interrater reliability assessments were conducted.

An additionally exemplary illustrations of the observational coding procedure are provided, see Additional File 1.

### Statistical analysis

A non-parametric Mann–Whitney-U-Test was used to determine whether there was a significant quantitative difference in the occurrence of aggressive episodes between the children with refugee experiences and children from clinical comparison group to explore qualitative differences in aggression categories between the groups. It was also employed to assess differences in the relation between trauma and aggression frequency between children with and without parent-reported traumatic experiences, independent on refugee status. The influence of environmental factors was investigated using Spearman's rho correlation, with parental education analysed both across the whole sample and separately for refugee and comparison group. All statistical analyses were done with SPSS 28.0 for Windows IBM [31].

## Results

### Participants

Of the 126 participants, 38% were girls. Because the proportion of boys was significantly higher in the comparison (72%) compared to comparison group (52%), the analyses were conducted separately for boys and girls. Children in the refugee group were mainly from Afghanistan (N = 51, 82%); 3 (5%) each from Palestine and from the Democratic Republic of Congo; and 1 (2%) each from Uganda, Bolivia, Venezuela, and Sierra Leone. See demographic and symptom characteristics of the children in Table 1.

**Table 1:** Demographic and symptom characteristics of the samples

	Refugee group				Comparison group			
	Boys	N	Girls	N	Boys	N	Girls	N
Mean age in years	5.23	32	4.78	30	5.23	46	5.09	18
Gender distribution (%)	51.6	32	48.4	30	71.9	46	28.1	18
Mean flight duration (in months)	33.3	29	29.5	25	–	–	–	–
Mean time since arrival in Germany (in months)	3.40	29	3.44	25	–	–	–	–
CATS								
At least one event/category reported	27	29	24	26	8	46	5	18
Mean number of events/categories reported	3.83	29	4.81	26	0.24	46	0.56	18
Mean number of symptoms	13.56	27	39.77	22	5.17	6	7.50	6
RHS								
Parental symptom load (symptoms)	27.64	28	34.09	23	7.58	46	7.97	18
Parental distress (thermometer score)	5.84	28	6.63	23	3.54	45	3.50	16
Mean years of parental education	5.71	32	6.86	30	11.44	46	12.66	18
Screen time								
< 1h	13	25	11	22	26	46	12	18
1–2h	2	25	5	22	10	46	6	18
> 2h	10	25	6	22	0	46	0	18

### Test results

#### H1a

 Episodes of aggressive behaviour in spontaneous play in children with and without refugee experiences.

**Fig. 1:**
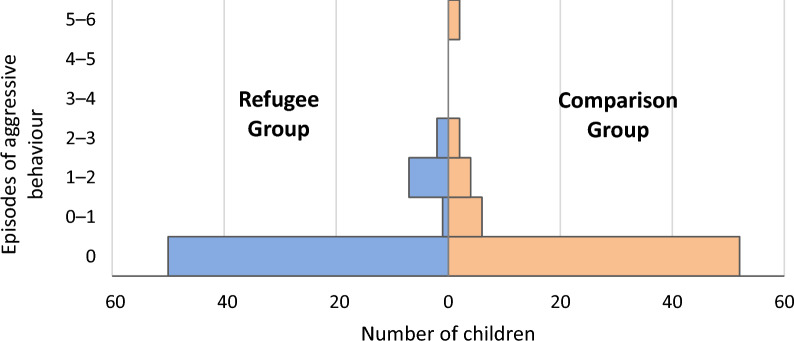
Episodes of aggressive behaviours during standardized play in children with and without refugee experiences

Aggressive behaviour sequences were observed in 10 out of 62 (16%) children with refugee experiences, for a total number of 16 episodes. In children without refugee experiences, aggressive behaviour sequences were observed in 14 out of 64 (22%) for a total of 26 episodes. The Mann–Whitney-U test revealed no significant difference in the distribution of aggressive episodes between the groups (U = 1887.000, Z = − 0.691, p = 0.490, r = 0.062, see Fig. 1). When stratified by gender, the difference still did not appear significant (for the boys p = 0.788, for the girls p = 0.268).

During observed aggressive episodes, children displayed different emotional states, usually playful, neutral, bored, and irritated. Compared to the clinical comparison group, where playful emotional states were the most frequent (45%), the refugee group showed fewer playful (31%) and more bored (31%), neutral (15%), and irritated (15%) emotional states.

#### H1b

Categories of aggressive behaviour.

**Fig. 2:**
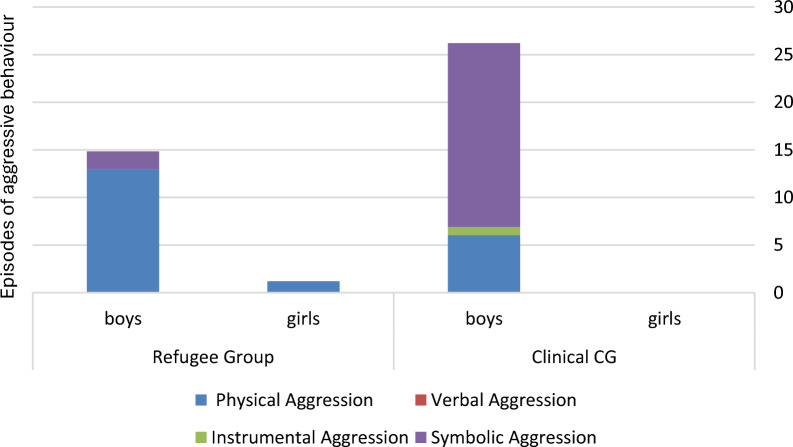
Distribution of different categories of aggressive behaviour among refugee and clinical comparison group, stratified for gender

For physical aggression (Category A), no significant differences were observed between the refugee and comparison group overall (p = 0.199) nor in either gender (for the boys p = 0.074, for the girls p = 0.439). Verbal Aggression (Category B) was not observed in either group. In the comparison group, only one child (2%) showed one sequence of instrumental aggression (Category C). For symbolic aggression (Category D), a difference was observed between the groups, with more symbolic aggression in the comparison group (U = 1706.500, Z =  − 2.565, p = 0.010, r = 0.229). After stratifying for gender, no such difference appeared among girls (U = 279.000, Z = 0.777, p = 0.439, r = 0.069), while the data suggest a group difference among boys, with more episodes of symbolic aggressive behaviour observed in boys from the comparison group (U = 583.500, Z = − 2.467, p = 0.014, r = − 0.219) (see Fig. 2).

#### H2

Relation of potentially traumatic experiences and aggression.

**Fig. 3:**
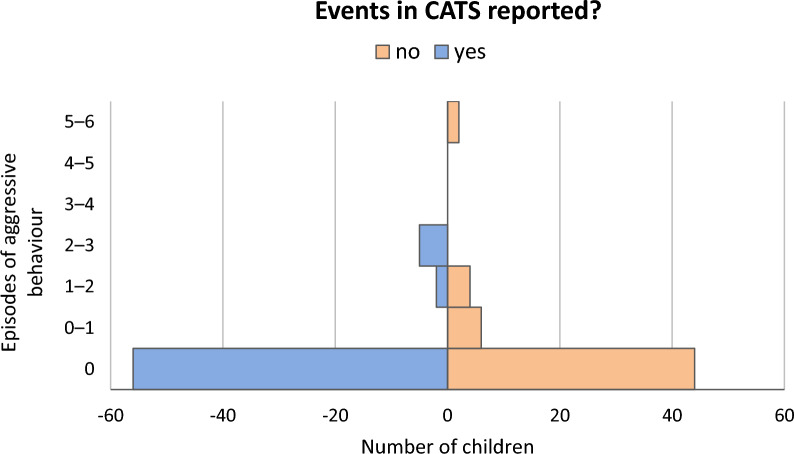
Events in CATS 3–6 and episodes of aggressive behaviour in children

For children with one or more events reported in the CATS 3–6 Questionnaire, we did not observe more aggressive behaviour episodes than for children with no reported events (U = 1559.000, Z = − 1.217, p = 0.224, r = − 0.112), as shown in Fig. 3. After accounting for gender, the test results remained non-significant (for the boys p = 0.478, for the girls p = 0.472).

After testing for each category of aggression, no group differences were found for physical, verbal, or instrumental aggression, whereas symbolic aggression was significantly higher in children without reported potentially traumatic experiences (U = 1532, Z = − 2.42,p  =  0.016, r = − 0.22). 

#### H3

Correlation between the number of episodes of aggressive behaviour and environmental factors.

There was no correlation between the number of episodes of aggressive behaviour and the flight duration or time since arrival in Germany for both gender groups. Additionally, the number of episodes of aggressive behaviour for the boys and for the girls did not correlate with the parents’ symptom load or stress. Independent of flight and trauma background, the correlation between the number of aggressive episodes and screen time was significant for the boys (r = 0.279, p = 0.018) and not significant for the girls, see Table 2. Additionally, mean years of parental education were not significantly associated with the frequency of aggressive expressions in play, neither in the pooled sample (ρ = 0.088, p = 0.401) nor when analysed separately for gender (see Table 2) and for the refugee (ρ = − 0.081, p = 0.615) and clinical comparison group (ρ = 0.119, p = 0.394).

**Table 2:** The number of episodes of aggressive behaviour in correlation with the total number of events in the CATS 3–6 Questionnaire, total number of symptoms in CATS 3–6, and environmental factors such as screen exposure, mental health of parents, months spent in Germany, duration of the flight, and total years of parental education stratified for gender

Spearman's rho	Episodes of aggressive behaviour			
	Boys		Girls	
	*N*	*r*	*N*	*r*
For the refugee group:				
Flight duration	29	0.211	25	0.085
Time in Germany	29	− 0.197	25	0.028
For the whole study sample:				
CATS 3–6:				
Events reported	75	− 0.065	44	− 0.055
Symptoms	33	0.165	28	0.322
Screen time	71	0.279*	40	− 0.133
RHS:				
Parental symptom load	74	0.129	41	0.254
Parental distress	73	0.062	39	0.188
Mean years of parental education	56	0.073	38	0.278

* p < 0.05, ** p < 0.001, Spearman-Correlation for all other variables

## Discussion

The present study investigated aggressive behaviours in children aged 3 to 6 years, focusing on how refugee and trauma experiences, as well as current environmental factors are related to their expression. For all children, we found only low occurrences of aggressive behaviour sequences during 10 min standardized playing sessions, independent of refugee and trauma experiences. Contrary to our hypothesis, we found no significant differences in amounts of aggressive episodes between children with and without refugee experience, even when accounting for gender. Further, our study identified significant differences in qualitatively observed categories of aggression, with a higher occurrence of symbolic aggression (e.g., fights with toys, shooting at animals) among boys from the comparison group. While no differences were found between children with and without reported potentially traumatic experiences in overall aggression or in physical, verbal, or instrumental aggression, a significant difference emerged for symbolic aggression, with higher levels observed among children for whom no potentially traumatic events were reported. Among environmental variables, instead of duration of flight or parental well-being (which are associated with childhood adversity), the amount of screen time showed a significant correlation with the number of observed episodes of aggressive behaviour in the male subgroup of our sample. During the study, emotional states accompanying aggressive behaviour were noted descriptively by the observer to provide context and background mood. Symbolic aggression in the comparison group was mainly accompanied by a playful and immersive emotional state, fitting within fantasy-driven play. In the refugee group, children's physical aggression was often paired with a bored, neutral, or irritated emotional state, suggesting less emotionally charged actions, potentially due to unfamiliarity with the materials or stress-related disengagement [23, 68].

Previous research indicates that greater exposure to Western toys and regular access to culturally typical play behaviour could promote fantasy-based conflicts, highlighting that symbolic expressions of aggression are shaped by different cultural practices and social learning instead of just reflecting aggressive tendencies [16, 69, 70]. Consistent with this perspective, the finding that symbolic aggression was more frequent among children without reported traumatic experiences further suggest that, rather than signalling emotional problems, symbolic aggression in our study appears to reflect a stimulating and developmentally conducive environment, where children feel secure enough to explore social dynamics through play. Consistent with earlier research [3, 40], our study highlights the importance of a better understanding of this form of play and recognizing it as a developmental and potentially beneficial aspect of early childhood.

Moreover, we conducted an analysis of some additional factors that may be associated with aggressive behaviour in children participating in our study. While longer flight duration, associated with more adversity for children, was expected to be a risk factor for increased aggression in general, spending more time in Germany might have made children more comfortable and used to Western toys, potentially leading to increased symbolic aggression. However, there was no correlation between flight duration or time in Germany and the number of episodes of aggressive behaviour, indicating that adaptation to the surroundings and toys is insufficient to explain the observed higher symbolic aggression in the comparison group.

Additionally, no correlation was found between a higher parental symptom load or increased distress level and more aggressive episodes in children. This finding supports reports in literature that many refugee children exhibit remarkable resilience despite caregiver psychopathology, particularly when their surroundings provide stability [40]. Interestingly, among boys in our study, higher screen time was associated with more observed aggressive behaviour. As we found a positive correlation between dysregulated screen exposure and biological stress levels in the clinical comparison group in our previous research [27], elevated stress likely negatively influences the ability to regulate impulses and to inhibit aggressive behaviour. In contrast, some longitudinal studies have found no effect of screen time on later aggression [71, 72], while other work has reported a correlation [73–75]. Two randomised trials suggested that reducing screen time can lower aggression, indicating a potential causal link between media consumption and aggressive behaviour [76, 77]. Finally, some studies yield mixed results on gender differences in the independent effect of screen time in terms of emotional and behavioural problems more broadly, some showing no differences between boys and girls [78], while others report a stronger effect on boys [75]. Another line of reasoning is based on the theoretical framework of observational learning, which suggests that children may imitate aggressive behaviour observed in real-life or in media content [35]. At the same time, previous research has demonstrated associations between higher overall screen exposure and behavioural and self-regulation difficulties in early childhood, even when media content was not differentiated [27]. On this basis, we consider it reasonable to cautiously interpret screen time quantity as an indicator of environmental exposure and daily routines. However, regarding the cross-sectional nature of this study, no statements can be made supporting an etiological link between aggression and higher screen exposure.

Rather than indicating what constitutes normative levels of aggression, the present findings highlight the strong context-dependence of expressions of aggressive behaviour in early childhood. When observed in a calm, non-violent, and low demand play situation, children with refugee experience as well as clinically referred children showed predominantly non-aggressive play behaviour, suggesting that safe and stimulative play settings promote peaceful playing behaviour. Finally, our findings underline the complexity in interpreting aggressive behaviour in young children, suggesting coexisting patterns of a developmentally meaningful form of play as well as potential reflection of difficulties in self-regulation. Temperament, understood as a value-neutral predisposition, may underlie both patterns, moderating how children respond to their environment [79]. In addition, differences in developmental maturity, language skills and abilities for symbolic play, self-regulatory capacities, socialization and co-regulation experiences, and momentary emotional states may further shape whether aggressive expressions emerge as playful exploration or as signs of regulatory challenge. Integrating this perspective allows us to interpret these behaviours not as inherently problematic, but as context-sensitive expressions of individual differences in reactivity and regulation.

### Strengths and limitations

The use of a clinically referred comparison group can be considered both, a strength and limitation of the present study. Children in this group were referred for developmental, language, or socio-emotional concerns, and therefore do not represent a population-based normative sample. Given consistent evidence linking various psychiatric diagnoses, to heightened impulsivity and aggressive behaviour in early childhood [80, 81], the observed levels of aggression may be inflated relative to those expected in non-clinical samples, which may limit the generalizability and influence interpretation of the findings. However, the sample with combined risk factors is frequently underrepresented in both public discourse and empirical research. By recruiting refugee and clinically referred families seeking support, the study avoids the common selection bias toward highly educated parent populations that often arises in studies based on voluntary participation [27, 82]. Additionally, Afghanistan was the origin for 82% of refugee children, limiting conclusions about other populations. At the same time, a substantial proportion of parents in the clinical comparison group had a migration background, resulting in overlapping contextual features across families and shared characteristics in both groups, like multilingual environments and experiences of relocation, allowing for a more valid comparison.

The operationalization of aggressive expressions in free individual play also represents both a limitation and a strength of our study. Aggressive behaviour linked to difficulties in self- or co-regulation is more likely to emerge in socially demanding or stressful situations, which were not elicited in this design. At the same time, observing children in a low-demand, non-provocative play context allows for the assessment of spontaneous aggressive expressions in the absence of external triggers and provides insight into children’s baseline tendencies to incorporate aggressive themes into play. Additionally, the 10 min play observation might not fully capture the complexity of children's behaviour, which can differ over time and across contexts [83, 84].

In addition, the observational coding of aggressive expressions was newly developed and has not yet undergone formal psychometric validation. All video material was coded by a single observer, and no quantitative interrater reliability measures were available. Observer bias can affect coding and interpretation, as cultural norms influence how behaviours are perceived; what seems aggressive in one culture may be seen as assertive in another [85, 86]. Furthermore, children’s emotional states during play were recorded descriptively and were not based on a standardized or validated coding system. This should be considered when interpreting our findings. Future studies should further validate this coding approach and include multiple observers.

Due to exploratory nature of our study, only the amount of children’s screen exposure was assessed, while we did not obtain any information about the content of the consumed media. As different types of media content may influence aggressive behaviour via observational learning mechanisms [35], future research should examine this issue.

While several effects reached statistical significance, effect sizes were small to moderate and should therefore be interpreted cautiously. Given the sample size, the study was primarily powered to detect moderate associations, and smaller effects may have remained undetected, limiting conclusions about non-significant findings. Additionally, although the RHS-15 showed excellent internal consistency in the current sample, its primary validation has focused on refugee populations, which should be considered when interpreting findings in mixed samples.

## Conclusion

While we expected flight-related and other adverse experiences of young children to be associated with expression of aggression during play, only the parent-reported amount of screen exposure correlated with the number of aggressive episodes among boys in the study. Overall, the findings highlight that aggressive expressions in early childhood are highly context-dependent, with both refugee and clinically referred sample showing predominantly non-aggressive play in calm, low-demand settings, underscoring the potential protective role of safe and stimulating play environments.

## Supplementary Information

Additional file 1.

## Data Availability

The anonymized dataset used and analysed during the current study are available from the corresponding author on reasonable request.
