# Supplementary material for: Aggressive behaviour during a standardized play observation in 3 to 6-year-old children with and without refugee experience: an observational study
Source: Child Adolesc Psychiatry Ment Health. 2026 Mar 27;20:54. doi: 10.1186/s13034-026-01071-y (PMC13063930; doi:10.1186/s13034-026-01071-y)
Supplement: Supplementary file 1 — Additional file 1. [file 13034_2026_1071_MOESM1_ESM.docx]

| **Investigated Child** | **Number of episodes** | **Category (A/B/C/D)** | **Emotional State** | **Comment** | **Video Duration** | **Corrected Number of episodes** |
| --- | --- | --- | --- | --- | --- | --- |
| a) | 1 | A | bored | Throws toys around | 13.24 | 0.75 |
| b) | 1 | A | neutral | Suddenly hits baby on the head without the context | 11.35 | 0.88 |
| c) | 2 | D | Immersed in play, hectic | Shoots on animals with a gun | 11.45 | 1.75 |
|  |  | C | neutral | Hits the house to close it |  |  |
| d) | 4 | D | Immersed in play | Overruns animals with the car | 7.4 | 5.4 |
|  |  | D | Immersed in play | Figures shoot each other |  |  |
|  |  | D | Immersed in play | A fight between animals and the man |  |  |

Supplementary Table 1: Exemplary coding of aggressive expressions observed during free individual play. Letters indicate the assigned aggression category (A = physical, C = instrumental, D = symbolic). The figure is intended to illustrate the coding procedure.
